# Mental Health Literacy in Young Adults: Adaptation and Psychometric Properties of the Mental Health Literacy Questionnaire

**DOI:** 10.3390/ijerph15071318

**Published:** 2018-06-23

**Authors:** Pedro Dias, Luísa Campos, Helena Almeida, Filipa Palha

**Affiliations:** 1 Faculty of Education and Psychology, Universidade Católica Portuguesa, Rua Diogo Botelho, 1327, 4169-005 Porto, Portugal; pdias@porto.ucp.pt (P.D.); fpalha@porto.ucp.pt (F.P.); 2 Research Center for Human Development, Rua Diogo Botelho, 1327, 4169-005 Porto, Portugal; 3 Faculty of Education and Psychology, Universidade Católica Portuguesa, Rua Diogo Botelho, 1327, 4169-005 Porto, Portugal; helenamfalmeida@hotmail.com; 4 ENCONTRAR+SE—Association for the Promotion of Mental Health, Rua Professor Melo Adrião 106, 4100-340 Porto, Portugal

**Keywords:** mental health literacy questionnaire, psychometric properties, young adults

## Abstract

Mental health literacy (MHL) is considered a prerequisite for early recognition and intervention in mental disorders, and for this reason, it has become a focus of research over the past few decades. Assessing this construct is relevant for identifying knowledge gaps and erroneous beliefs concerning mental health issues, to inform the development of interventions aimed at promoting mental health literacy as well as the evaluation of these interventions. Recently, we developed a new self-reporting measure (MHLq) for assessing mental health literacy in young people (12–14 years-old), meeting the need to assess MHL from a comprehensive perspective of the construct instead of focusing on a restricted number of mental disorders or specific dimensions (e.g., knowledge concerning specific disorders; stigma). The present study aimed to adapt the MHLq for the young adult population and to examine its psychometric properties, according to the following steps: (1) item adaptation, using a think aloud procedure (n = 5); (2) data collection (n = 356, aged between 18 and 25 years old; and (3) psychometric analyses (exploratory factor analysis and internal consistency analysis). The final version of the questionnaire included 29 items (total scale α = 0.84), organized by four dimensions: (1) knowledge of mental health problems (α = 0.74); (2) erroneous beliefs/stereotypes (α = 0.72); (3) help-seeking and first aid skills (α = 0.71); and (4) self-help strategies (α = 0.60). The results suggest that the MHLq-adult form is a practical, valid, and reliable screening tool for identifying gaps in knowledge, beliefs, and behavioral intentions related to mental health and mental disorders, planning promotion programs, and evaluating intervention effectiveness.

## 1. Introduction

Mental health literacy, a concept introduced by Jorm and colleagues [1], was first defined as “knowledge and beliefs about mental disorders which aid their recognition, management or prevention” (p. 182). A recent update on this concept included the ability to provide support to someone presenting with a mental health problem, that is, first aid skills [2]. Accordingly, mental health literacy is not limited to having knowledge as knowledge is linked to beliefs that together determine attitudes (e.g., resistance to seek professional help). 

The assessment of knowledge and beliefs related to mental health problems allows for the identification of stigma associated to those problems, which is considered to be one of the main barriers to early recognition and intervention [3]. Furthermore, assessing the knowledge gaps and erroneous beliefs concerning mental health issues enables the development of interventions aimed at promoting mental health literacy [4,5] as well as the evaluation of these interventions.

Several instruments have been developed to assess mental health literacy, the stigma associated with mental health problems, and the related constructs, e.g., the interview used for the Australian national survey of youth and parents [6]; the questionnaire used to evaluate the program, “Crazy? So what!” [4]; the instrument developed under the scope of the Mind Matters project [7]; the Attribution Questionnaire [8]; The Attitudes to Mental Illness Questionnaire [9]; and the Mental Health Knowledge Schedule, MAKS [10]. As we have pointed out in a previous article [11], most of the available instruments either assess specific dimensions of mental health literacy (e.g., knowledge; stigmatizing perceptions) or specific mental health problems or diagnoses (e.g., schizophrenia; depression). Taking into account the updated construct of MHL, and the abovementioned limitations of previous measures, there is a need for new instruments to provide a more up-to-date assessment of this construct. 

Recently, we developed a new self-reporting measure (MHLq) for assessing mental health literacy in young people (12–14 years-old), meeting the need to assess it from a comprehensive perspective of the construct instead of focusing on a restricted number of mental disorders or specific dimensions. The questionnaire includes 33 items, organized in three subscales: first aid skills and help seeking; knowledge/stereotypes; and self-help strategies. The MHLq showed good internal consistency and excellent test-retest reliability. It is a practical, valid, and reliable tool for identifying gaps in knowledge, beliefs, and behavioral intentions in large samples, allowing the development and evaluation of interventions aimed at promoting mental health in young people [11].

**Figure 1:**
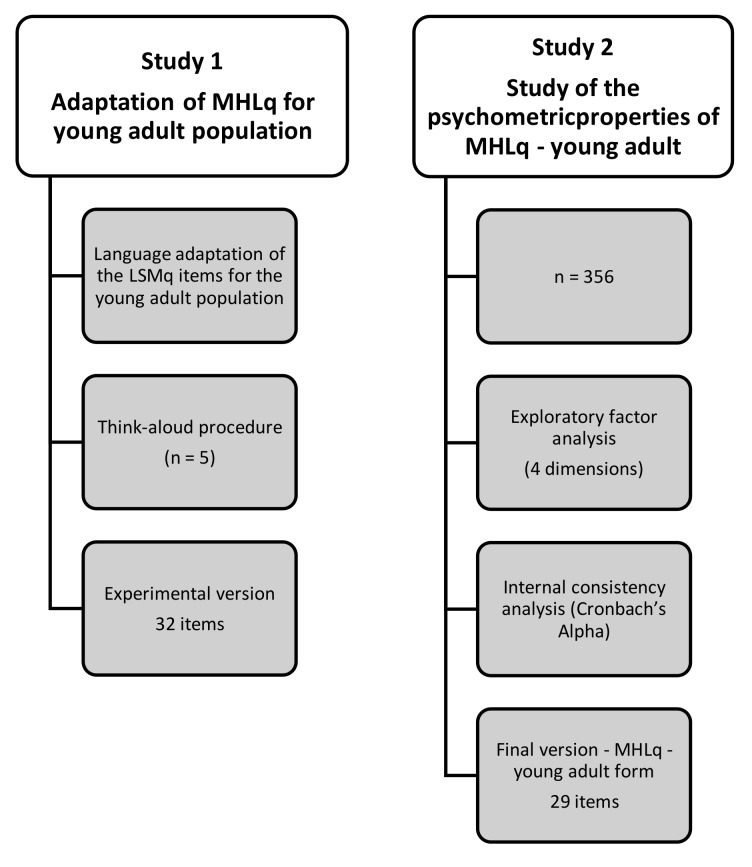
Development of adaptation and study of psychometric properties of the mental health literacy questionnaire (MHLq)-young adult.

This article presents the process of adapting the MHLq for young adults and the study of its psychometric properties through two studies. Study 1 looked at the adaptation of the MHLq for the young adult population, and Study 2 looked at the psychometric properties of the MHLq-young adult form. Figure 1 presents a flowchart of the study design.

## 2. Study 1: Adaptation of MHLq-Young Adult Population 

Study 1 followed two stages: (1) language adaptation of the MHLq [11] items to the young adult population; and (2) the think-aloud procedure.

### 2.1. Language Adaptation of the MHLq Items to the Young Adult Population

The research team revised the phrasing of both the socio-demographic form and the MHLq items (since the questionnaire was developed for a target population of young people) by replacing it by more formal speech (e.g., in the sociodemographic form, the question “Are you a girl?” in the MHLq was changed to “Gender” in the MHLq-young adults form), and other questions were also added (e.g., marital status, academic qualifications, and profession/occupation).

### 2.2. Think-Aloud Procedure 

The second stage of the adaptation of the MHLq was a think-aloud procedure with a group of five participants aged between 22 and 35 years old; two females and three males; four postgraduate students and one professional technician in the area of multimedia. Informed consent was given by all participants. All of the 32 items were presented individually to this group, and participants were asked to comment on the items based on item interpretation and suggested rephrasing for increasing their suitability for the target age group. All comments and suggestions made by the participants were registered by the research team. 

### 2.3. Results

Based on the team’s revision and the think-aloud procedure, 15 items were rephrased, two items were removed from the questionnaire because their content was redundant when compared to another item, and two items were added to the young adult form, replacing one item in the original form. Table 1 presents the changes from the MHLq to the experimental version of the MHLq-young adult form.

## 3. Study 2: Study of the Properties of MHLq-Young Adults Form

### 3.1. Materials and Methods

#### 3.1.1. Participants

The experimental version of the MHLq-young adults form was administered to a group of 356 participants aged between 18 and 25 years old (M = 21.13; SD = 3.69). Of the young adults, 47% were male, 97.5% were single, and 97.2% were Portuguese. Most participants (88.6%) were students, attending college (n = 214) and other adult training programs in professional schools (n = 89). The non-students participants’ (n = 35) educational attainment was predominantly secondary education (n = 30) (see Table 2).

A total of 159 participants (44.7%) reported knowing someone who had a mental health problem, 160 students (44.9%) stated they did not know anyone with these problems, and 37 (4%) were not aware of anyone with these problems. Regarding the degree of proximity, it was mentioned most often as being friends (n = 48; 30.2%).

#### 3.1.2. Measures

The experimental version of the questionnaire resulting from Study 1 included: (1) a socio-demographic form comprised of questions related to the participants’ gender, age, marital status, nationality, residence, academic qualifications, profession/occupation (if they were a student, they indicated the school grade), proximity to people with mental health problems including the nature of the relationship; and (2) 32 items, organized in a 5-point Likert response scale (1 = strongly disagree; 2 = disagree; 3 = neither agree nor disagree; 4 = agree; and 5 = strongly agree).

### 3.2. Procedures

#### 3.2.1. Data Collection

Data collection followed ethical guidelines with all participants signing an informed consent form. The sociodemographic form and the experimental version of the MHLq-young adults form were self-administered to participants in their educational or work environments. In order to increase the number of non-students, a snowball sampling procedure was implemented.

#### 3.2.2. Analytic Plan

Psychometric properties, i.e., the construct validity and internal consistency of the MHLq-young adults form were evaluated using exploratory factor analysis (principal components analysis, with Varimax rotation) and Cronbach’s Alpha. Both analyses were used as complementary procedures for determining the final structure of the instrument [12]. The criteria used for the factor analysis were: (a) item loadings larger or equal to 0.20; and (b) the content of items loading in factors should be compatible with the underlying theoretical content. 

Descriptive statistics were used to characterize the participants’ mental health literacy levels, and the overall scores (sum of the values of all the items) and scores by factors (sum of the values of the dimension items). Higher values in all dimensions and the total MHLq score corresponded to higher levels of mental health literacy. For that reason, six items had to be reverse-coded.

The relationship between mental health literacy levels and sociodemographic variables (gender and proximity to mental health problems) was also explored, using *t*-tests for independent samples.

In all hypothesis tests a level of significance of α = 5% was considered.

The analysis was performed using the statistical analysis program IBM SPSS Statistics^®^ v.22.0 (IBM Inc., Armonk, NY, USA).

### 3.3. Results

The first exploratory factor analysis, retaining all factors with an eigenvalue higher than 1.0 failed to meet the conceptual organization of the instrument.

Therefore, new exploratory factor analyses, using a fixed number of factor extraction procedures (three factors based on the original version of the MHLq, and four factors based on the conceptual option) were conducted. The four-factor structure was the best solution in terms of explained variance and conceptual item loading. 

The final factor structure included: (1) items related to knowledge of mental health problems; (2) items related to erroneous beliefs/stereotypes; (3) items related to first aid skills and help seeking behavior; and (4) items related to self-help strategies. This solution was responsible for 36.99% of the variance. Three items were removed from the questionnaire: one did not load in any of the factors and two did not load in the corresponding conceptual dimension.

Table 3 presents the factorial structure of the MHLq-young adult form including the 29 items that were maintained.

Cronbach’s Alpha values were good for the total score, ranging from acceptable to questionable for the subscales: Total Score (29 items) α = 0.84; Factor 1, knowledge of mental health problems (11 items) α = 0.74; Factor 2, erroneous beliefs/stereotypes (eight items) α = 0.72; Factor 3, first aid skills and help seeking behavior (six items) α = 0.71; Factor 4, self-help strategies (four items) α = 0.60. Factor 4, which presented the lowest Alpha value, includes only four items. Therefore, the item-total correlation for this subscale was analyzed. Results ranged between 0.29 and 0.49, and the removal of any of the subscale items would result in a decrease in Alpha value.

The total score for the 29 items of the MHLq-young adults form ranged between 29 and 145 (M = 105.27; SD = 7.05). The knowledge of mental health problems factor scores ranged between 11 and 55 (M = 44.50; SD = 4.45). Erroneous beliefs/stereotypes factor scores—six of the eight items were reverse-scored (items 6, 10, 13, 15, 23, and 27)—ranged between 8 and 40 (M = 19.75; SD = 2.92). The first aid skills and help seeking behavior factor ranged between 6 and 30 (M = 24.13; SD = 3.33). The self-help strategies factor scores ranged between 4 and 20 (M = 16.90; SD = 1.90).

Socio-demographic variables (gender and proximity with people presenting with mental disorders) are related to differences in MHLq scores (see Table 4 and Table 5). Regarding gender differences, females showed higher scores than males on the MHLq global score and in all dimensions, except for Erroneous beliefs/stereotypes, in which no significant differences were found. Participants who indicated knowing someone with a mental health problem showed higher scores than participants who did not know anyone presenting such problems, on the MHLq total score, knowledge of mental health problems, self-help strategies, and low erroneous beliefs/stereotypes dimension.

## 4. Discussion

The likelihood of most individuals developing a mental health problem is high, as demonstrated by the significant increase in the prevalence of mental health problems throughout the lifespan [3,13]. Increasing levels of mental health literacy contributes to the promotion of mental health and may play an important role in early identification and intervention when a psychological/mental health problem develops [2,13].

The present study aimed to adapt and examine the psychometric properties of a brief self-report questionnaire designed to assess mental health literacy in young adults, based on a previous measure developed for young people [11].

The experimental version of the MHLq-young adult form was applied to a sample of 356 participants. Construct validity, assessed by exploratory factor analysis, revealed a four-dimension factorial structure of the MHLq-young adult form, consistent with the multidimensional perspective of the construct of mental health literacy [2]. Internal consistency, assessed with Cronbach’s Alpha, showed acceptable to good reliability scores for three of the questionnaire’s dimensions and global score. The lower alpha of the Self-help strategies subscale (α = 0.60) could be explained by the scale’s low number of items (c.f. [14]). Nevertheless, the item-total correlation of its four items, as well as the fact that item deletion procedures would not result in higher internal consistency scores, supported the decision to keep this subscale in this first version of the instrument. Future research should reassess internal consistency scores of this subscale and, if scores remain low, its revision should be considered.

This study explored the impact of sociodemographic variables on the levels of mental health literacy. Differences were found regarding gender and proximity with someone with a mental health problem. In general, females and participants who reported knowing someone with a mental health problem showed higher levels of mental health literacy, in line with previous research [8,11,15,16,17,18,19].

Future research should expand the study of the psychometric properties of the MHLq-adults form with larger and representative samples of the adult population. The sample of the present study included, mostly, university level students, with a high degree of familiarity with education and elaborated language. The use of a more heterogeneous sample would allow testing the items language appropriateness with lower-educated individuals, as well as to examine Mental Health Literacy levels in the adult population. Furthermore, other analyses should include more sophisticated psychometric analyses, based on Structural Equation Modelling procedures, such as confirmatory factor analysis, and convergent and discriminant validity procedures. The evolution of the Mental Health Literacy construct, already displayed in this instrument through the inclusion of items related to self-help strategies may also be subject to new studies, focusing on the positive MHL, as suggested by recent works (e.g., [20]).

## 5. Conclusions

The Mental Health Literacy questionnaire-young adults form overcomes several limitations of other MHL assessment instruments, which include an exclusive focus on specific MHL dimensions and/or a restricted number of mental disorders and time-consumption. It does this by providing a short, valid and reliable self-report assessment, based on a comprehensive approach to this construct, including knowledge about mental health problems, erroneous beliefs/stereotypes, first-aid skills and help seeking behavior, and self-help strategies.

Mental health professionals and researchers may use this measure for designing and evaluating mental health literacy promotion programs, and as a screening tool for the identification of intervention needs in the young adult population in different settings, for example in the work environment and in higher education organizations. Since this instrument was developed in the Portuguese language, the use of MHLq-young adult form in other languages should be preceded by its translation, back translation and validation to local culture, and psychometric study.

## Figures and Tables

**Table 1:** MHLq items, MHLq-young adult version Portuguese and English version.

Mhlq	Mhlq-Young Adult (Original Version in Portuguese)	Mhlq-Young Adult (English Version)
If a friend of mine developed a mental disorder, I would offer her/him support.	Se uma pessoa próxima de mim estivesse com uma perturbação mental, oferecia-me para ajudar.	If someone close to me had a mental disorder, I would offer him/her help.
Physical exercise helps to improve mental health.	A prática de exercício físico contribui para uma boa saúde mental.	Physical exercise contributes to good mental health.
A person with depression feels very miserable.	Uma pessoa com depressão sente-se muito infeliz.	A person with depression feels very miserable (unchanged item).
People with schizophrenia usually have delusions (e.g., they may believe they are constantly being followed and observed).	Em casos de esquizofrenia, é comum as pessoas terem ideias delirantes (p. ex., podem acreditar que estão a ser constantemente seguidas e observadas).	People with schizophrenia usually have delusions (e.g., they may believe they are constantly being followed and observed) (unchanged item).
If I had a mental disorder I would seek my family’s help.	Se eu estivesse com uma perturbação mental, procuraria ajuda de pessoas da minha família.	If I had a mental disorder I would seek my relatives’ help.
If a friend of mine developed a mental disorder, I would encourage her/him to look for a psychologist.	Se uma pessoa próxima de mim estivesse com uma perturbação mental, eu encorajava-a a procurar um psicólogo.	If someone close to me had a mental disorder, I would encourage her/him to look for a psychologist.
Mental disorders don’t affect people’s behaviors.	Uma perturbação mental não afeta o comportamento.	Mental disorders don’t affect people’s behaviors (unchanged item).
If a friend of mine developed a mental disorder, I would talk to her/his parents.	-	Removed
Good sleep helps to improve mental health.	Dormir bem contribui para uma boa saúde mental.	Sleeping well contributes to good mental health.
If I had a mental disorder I would seek professional help (psychologist and /or psychiatrist.	Se eu estivesse com uma perturbação mental, procuraria a ajuda de um psicólogo. Se eu estivesse com uma perturbação mental, procuraria a ajuda de um médico psiquiatra.	If I had a mental disorder I would seek a psychologist’s help (new item). If I had a mental disorder I would seek a psychiatrist’s help (new item).
A person with anxiety disorder may panic in situations that she/he fears.	Uma pessoa com perturbação de ansiedade pode entrar em pânico perante situações de que tenha medo.	A person with anxiety disorder may panic in situations that she/he fears (unchanged item).
People with mental disorders come from families with little money.	As pessoas com perturbação mental são de famílias com baixos recursos económicos.	People with mental disorders belong to low-income families.
If a friend of mine developed a mental disorder, I would listen to her/him without judging or criticizing.	Se uma pessoa próxima de mim estivesse com uma perturbação mental, eu ouvia-a sem julgar ou criticar.	If someone close to me had a mental disorder, I would listen to her/him without judging or criticizing.
Alcohol use may cause mental disorders.	O consume de álcool pode causar perturbações mentais.	Alcohol use may cause mental disorders (unchanged item).
Mental disorders don’t affect people’s feelings.	Uma perturbação mental não afeta os sentimentos.	Mental disorders don’t affect people’s feelings (unchanged item).
The sooner mental disorders are identified and treated, the better.	Quanto mais cedo forem identificadas e tratadas as perturbações mentais, melhor.	The sooner mental disorders are identified and treated, the better (unchanged item).
Only adults have mental disorders.	Só os adultos têm perturbações mentais.	Only adults have mental disorders (unchanged item).
Brain malfunctioning may cause the development of mental disorders.	Alterações no funcionamento cerebral podem levar ao aparecimento de perturbações mentais.	Changes in brain function may lead to the onset of mental disorders.
If a friend of mine developed a mental disorder, I would encourage her/him to get medical support.	Se uma pessoa próxima de mim estivesse com uma perturbação mental, eu encorajava-a a procurar um médico psiquiatra.	If someone close to me had a mental disorder, I would encourage her/him to see a psychiatrist.
If I had a mental disorder I would seek my friends’ help	Se eu estivesse com uma perturbação mental procuraria a ajuda de amigos.	If I had a mental disorder I would seek friends’ help.
Having a balanced diet helps to improve mental health.	Uma alimentação equilibrada contribui para uma boa saúde mental.	A balanced diet contributes to good mental health.
One of the symptoms of depression is the loss of interest or pleasure in most things.	Um dos sintomas da depressão é a falta de interesse ou prazer pela maioria das coisas.	One of the symptoms of depression is the loss of interest or pleasure in most things (unchanged item).
A person with anxiety disorder avoids situations that may cause her/him distress.	-	Removed
If a friend of mine developed a mental disorder, I wouldn’t be able to help her/him.	Se uma pessoa próxima de mim estivesse com uma perturbação mental, eu não poderia fazer nada para a ajudar.	If someone close to me had a mental disorder, I could not be of any assistance.
The symptom’s length is one of the important aspects to determine whether a person has or does not have a mental disorder.	A duração dos sintomas é um dos critérios importantes para o diagnóstico de uma perturbação mental.	The symptom’s length is one of the important criteria for the diagnosis of a mental disorder.
Depression is not a true mental disorder.	A depressão não é uma verdadeira perturbação mental.	Depression is not a true mental disorder (unchanged item).
Drug addiction may cause mental disorders.	O consumo de drogas pode causar perturbações mentais.	Drug addiction may cause mental disorders (unchanged item).
Mental disorders affect people’s thoughts.	Uma perturbação mental afeta os pensamentos.	Mental disorders affect people’s thoughts (unchanged item).
If a friend of mine developed a mental disorder, I would talk to the form teacher or other teacher.	-	Removed.
Doing something enjoyable helps to improve mental health.	Fazer algo que dê prazer contribui para uma boa saúde mental.	Doing something enjoyable contributes to a good mental health.
A person with schizophrenia may see and hear things that nobody else sees and hears.	Uma pessoa com esquizofrenia pode ver e ouvir coisas que mais ninguém vê e ouve.	A person with schizophrenia may see and hear things that nobody else sees and hears (unchanged item).
Talking over problems with someone helps to improve mental health.	Falar sobre os problemas pessoais com alguém próximo contribui para uma boa saúde mental.	Talking over problems with someone close to me contributes to a good mental health.
Highly stressful situations may cause mental disorders.	Situações de grande stress podem causar perturbações mentais.	Highly stressful situations may cause mental disorders (unchanged item).

**Table 2:** Sociodemographic characteristics of the sample.

Sociodemographic Variables	n	Valid %
Gender		
Female	186	53
Male	165	47
Mean age (SD)	21.13	(3.69)
Occupation		
Studying in college/university	214	63.3
Studying in adult training programs	89	26.3
Working	35	10.4
Marital Status		
Single	346	97.5
Married	9	2.5
Nationality		
Portuguese	345	97.2
Other	10	2.8
Working	35	10.4

**Table 3:** Exploratory factor analysis of the MHLq-young adult form item description and factor loadings (final version, 29 items).

MHLq-Young Adult Form Items	Factor 1	Factor 2	Factor 3	Factor 4
	Knowledge of Mental Health Problems	Erroneous Beliefs/Stereotypes	First Aid Skills and Help Seeking Behaviour	Self-Help Strategies
25. Mental disorders affect people’s thoughts.	**0.587**			
27. A person with schizophrenia may see and hear things that nobody else sees and hears.	**0.580**			
24. Drug addiction may cause mental disorders.	**0.561**			
16. Changes in brain function may lead to the onset of mental disorders.	**0.552**			0.294
28. Highly stressful situations may cause mental disorders.	**0.552**			
3. People with schizophrenia usually have delusions (e.g., they may believe they are constantly being followed and observed).	**0.516**			
12. Alcohol use may cause mental disorders.	**0.482**			
22. The symptom’s length is one of the important criteria for the diagnosis of a mental disorder.	**0.464**	0.299		
26. Doing something enjoyable contributes to a good mental health.	0.428			**0.347**
20. One of the symptoms of depression is the loss of interest or pleasure in most things.	**0.424**	0.308		
23. Depression is not a true mental disorder.	−0.285	**−0.274**		
10. People with mental disorders belong to low-income families.		**−0.707**		
15. Only adults have mental disorders.		**−0.658**		
6. Mental disorders don’t affect people’s behaviors.		**−0.607**		
14. The sooner mental disorders are identified and treated, the better.		**0.574**	0.213	0.248
13. Mental disorders don’t affect people’s feelings.	−0.232	**−0.568**		
9. A person with anxiety disorder may panic in situations that she/he fears.	**0.349**	0.507	0.204	
11. If someone close to me had a mental disorder, I would listen to her/him without judging or criticizing.		**0.391**		0.350
29. If I had a mental disorder I would seek a psychiatrist’s help.			**0.745**	
17. If someone close to me had a mental disorder, I would encourage her/him to see a psychiatrist.			**0.710**	
8. If I had a mental disorder I would seek a psychologist’s help.			**0.661**	
4. If I had a mental disorder I would seek my relatives’ help.			**0.630**	
5. If someone close to me had a mental disorder, I would encourage her/him to look for a psychologist.		0.511	**0.516**	
2. A person with depression feels very miserable.	**0.227**		0.291	
19. A balanced diet contributes to good mental health.	0.308			**0.634**
1. Physical exercise contributes to good mental health.	0.204			**0.563**
7. Sleeping well contributes to good mental health.	0.203	0.260		**0.541**
21. If someone close to me had a mental disorder, I could not be of any assistance.		**−0.220**		−0.488
18. If I had a mental disorder I would seek friends’ help.			**0.403**	0.453
R^2^ (%)	10.86	10.32	8.59	7.22

Note: loadings in bold represent items retained for each factor.

**Table 4:** Gender differences in mental health literacy (MHLq global score and dimensions).

	Gender		
	Male	Female	
Mental health literacy (global score)	(n = 153) Mean (sd)	(n = 174) Mean (sd)	*t*(325)
	103.93 (7.10)	106.37 (6.89)	−3.15 **
Knowledge of mental health problems	(n = 158) Mean (sd)	(n = 181) Mean (sd)	*t*(337)
	43.71 (4.44)	45.16 (4.37)	−3.03 **
Erroneous beliefs/stereotypes	(n = 159) Mean (sd)	(n = 181) Mean (sd)	*t*(338)
	20.03 (3.07)	19.45 (2.75)	1.85
First aid skills and help seeking behavior	(n = 163) Mean (sd)	(n = 182) Mean (sd)	*t*(343)
	23.63 (3.46)	24.53 (3.20)	−2.52 *
Self-help strategies	(n = 163) Mean (sd)	(n = 182) Mean (sd)	*t*(343)
	23.63 (3.46)	24.53 (3.20)	−2.52 *

* *p* < 0.05; ** *p* < 0.01.

**Table 5:** Differences in mental health literacy (MHLq global score and dimensions) based on proximity to people with mental health problems.

	Proximity to People with Mental Health Problems		
	Yes	No	
Mental health literacy (global score)	(n = 149) Mean (sd)	(n = 152) Mean (sd)	*t*(299)
	106.62 (7.04)	104.34 (6.62)	−2.90 **
Knowledge of mental health problems	(n = 156) Mean (sd)	(n = 155) Mean (sd)	*t*(309)
	45.81 (4.48)	43.48 (3.98)	−4.85 ***
Erroneous beliefs/stereotypes	(n = 157) Mean (sd)	(n = 152) Mean (sd)	*t*(307)
	19.99 (2.71)	19.22 (2.80)	−2.46 *
First aid skills and help seeking behavior	(n = 156) Mean (sd)	(n = 158) Mean (sd)	*t*(312)
	24.17 (3.64)	24.31 (2.95)	0.38
Self-help strategies	(n = 159) Mean (sd)	(n = 160) Mean (sd)	*t*(317)
	17.28 (1.75)	16.66 (1.97)	−2.98 **

* *p* < 0.05; ** *p* < 0.01; *** *p* < 0.001.
